# Glucagon-like peptide-1 enhances cardiac L-type Ca^2+ ^currents via activation of the cAMP-dependent protein kinase A pathway

**DOI:** 10.1186/1475-2840-10-6

**Published:** 2011-01-20

**Authors:** Yong-Fu Xiao, Alena Nikolskaya, Deborah A Jaye, Daniel C Sigg

**Affiliations:** 1 Cardiac Rhythm Disease Management, Medtronic, Inc., 8200 Coral Sea Street NE, Mounds View, MN 55112, USA

## Abstract

**Background:**

Glucagon-like peptide-1 (GLP-1) is a hormone predominately synthesized and secreted by intestinal L-cells. GLP-1 modulates multiple cellular functions and its receptor agonists are now used clinically for diabetic treatment. Interestingly, preclinical and clinical evidence suggests that GLP-1 agonists produce beneficial effects on dysfunctional hearts via acting on myocardial GLP-1 receptors. As the effects of GLP-1 on myocyte electrophysiology are largely unknown, this study was to assess if GLP-1 could affect the cardiac voltage-gated L-type Ca^2+ ^current (I_Ca_).

**Methods:**

The whole-cell patch clamp method was used to record I_Ca _and action potentials in enzymatically isolated cardiomyocytes from adult canine left ventricles.

**Results:**

Extracellular perfusion of GLP-1 (7-36 amide) at 5 nM increased I_Ca _by 23 ± 8% (*p *< 0.05, n = 7). Simultaneous bath perfusion of 5 nM GLP-1 plus 100 nM Exendin (9-39), a GLP-1 receptor antagonist, was unable to block the GLP-1-induced increase in I_Ca_; however, the increase in I_Ca _was abolished if Exendin (9-39) was pre-applied 5 min prior to GLP-1 administration. Intracellular dialysis with a protein kinase A inhibitor also blocked the GLP-1-enhanced I_Ca_. In addition, GLP-1 at 5 nM prolonged the durations of the action potentials by 128 ± 36 ms (*p *< 0.01) and 199 ± 76 ms (*p *< 0.05) at 50% and 90% repolarization (n = 6), respectively.

**Conclusions:**

Our data demonstrate that GLP-1 enhances I_Ca _in canine cardiomyocytes. The enhancement of I_Ca _is likely via the cAMP-dependent protein kinase A mechanism and may contribute, at least partially, to the prolongation of the action potential duration.

## Background

Glucagon-like peptide-1 (GLP-1) is one of the transcription products from the proglucagon gene. GLP-1 is a peptide hormone predominately produced by intestinal endocrine L-cells. GLP-1 has two major isoforms (7-36 amide and 7-37) which are among the most potent stimulators of glucose-dependent insulin secretion. Both peptides are considered equipotent in terms of their biological activity[1,2]. GLP-1 stimulates glucose-dependent insulin secretion and insulin biosynthesis and inhibits glucagon secretion, gastric emptying, and food intake. The N-terminal degradation of GLP-1 by dipeptidyl peptidase-4 (DPP-4)-mediated cleavage at the position 2 alanine modifies its biological activity[3].

GLP-1 modulates multiple cellular functions believably via acting on GLP-1 receptors (GLP-1Rs)[4], which are expressed in the human pancreas, heart, lung, kidney, stomach and brain[5]. Due to the multiple beneficial effects of GLP-1R agonists in the treatment of diabetes mellitus including weight loss, pharmaceutical companies have developed and introduced GLP-1R agonists as a treatment option for patients with type 2 diabetes mellitus (e.g. Byetta™ (exenatide) and Victoza™ (liraglutide)). Various cardiovascular effects of GLP-1 have been reported. GLP-1 infusion improves glucose uptake[6] and metabolism[7], as well as cardiac function[6] and hemodynamics[8-10] in different species, including in humans[11]. In addition, GLP-1 infusion reduced infarct size in a rodent model of ischemia with no effect on ventricular function[12,13], but did not alter infarct size in an open-chest, anesthetized porcine model of ischemia[5]. Furthermore, GLP-1R knockout mice (GLP-1R^-^/^-^) have lower heart rate and blood pressure with an increase in cardiac mass[14]. In clinical studies using liraglutide (Victoza™), a GLP-1R agonist, a consistent decrease in blood pressure and a reduction of cardiovascular risk markers were observed in a cohort of over 5000 patients[15].

In pancreatic β-cells, GLP-1 inhibits ATP-dependent K^+ ^channels via the cAMP-mediated PKA pathway[16-19]. The effects of GLP-1 on myocardium observed in several studies are potentially via its G protein-coupled receptors[10,20]. Recently, a new glucagon-like peptide isolated from the intestine of the eel, *Anguilla japonica*[21] with a structure similar to that of oxyntomodulins has shown an inotropic effect via stimulation of Ca^2+ ^influx and a chronotropic effect independent of extracellular Ca^2+^. In addition, GLP-1 and glucose-dependent insulinotropic polypeptide can enhance β-cell cytoplasmic Ca^2+ ^oscillation and increase insulin secretion via activation of cAMP-triggered cascades[22,23]. However, the effects of GLP-1 on myocyte electrophysiology have not been carefully assessed. Therefore, in the present study, we investigated the effects of GLP-1 on the voltage-gated L-type Ca^2+ ^channel in isolated canine left ventricular myocytes.

## Methods

The study was designed and conducted in accordance with the *Guide for the Care and Use of Laboratory Animals *[Department of Health and Human Services Publication No. (NIH) 85-23, Revised 1996]. The animal protocol was approved by the Institutional Animal Care and Use Committee of the Physiological Research Laboratories of Medtronic, Inc.

### Isolation of canine left ventricular myocytes

Single left ventricular myocytes were enzymatically isolated from adult canine hearts with a body weight of 30 to 33 kg (n = 13) by the methods described previously[24]. Briefly, left ventricular heart tissue was rapidly excised from canine hearts. Several tissue pieces weighing 3-5 grams each were harvested from the left ventricle and immediately placed in ice-cold Ca^2+^-free dissection buffer ("cardioplegia") solution containing (mM): NaCl 140, KCl 5.4, MgCl_2 _1.2, HEPES 5, glucose 5.5, and 2,3-butanedione monoxime 30, pH 7.4. Tissue pieces were rinsed twice by the dissection solution supplemented with antibiotics and cut into small pieces (~1 mm^3^) with sharp scissors. Digestion buffer solution was aspirated and replaced with a Ca^2+^-free enzymatic solution containing 0.1% trypsin, 0.075% collagenase type II, 0.05% cyaluronidase, and 0.025% elastase. Tissue was incubated in Ca^2+^-free enzymatic solution at 4°C overnight and then subjected to a 37°C digestion procedure.

During 37°C digestion, Ca^2+^-free enzymatic solution containing trypsin was gradually replaced with another enzymatic solution containing 0.1 mM of Ca^2+^, 0.075% collagenase, 0.1% soybean trypsin inhibitor, and 0.2% bovine serum albumin. The tissues were agitated in a heated shaker (~300 rpm at 37°C) for several 15-min intervals. After agitation, the tissue was triturated about 15-20 times with a 10 ml pipette. The tissue was allowed to settle, supernatants were removed, and a fresh aliquot of enzymatic solution was added. The first two supernatants containing mostly cell debris were aspirated and discarded. The subsequent supernatants containing liberated cells were collected into centrifuge tubes, where a cell culture media with 20% fetal bovine serum was added. Collected cell suspensions were centrifuged for 5 min at 300 rpm and the resulting supernatant was discarded. The myocyte pellets were resuspended in a HEPES-buffered cell culture media. Yields from this procedure were about 50% of Ca^2+^-tolerant myocytes. Quiescent, rod-shaped ventricular myocytes with clear striations were chosen for patches to study their electrophysiology and GLP-1 effects.

### Electrophysiological recordings

After dissociation, a small amount (30 μl) of the medium solution with myocytes was transported to a chamber mounted on the stage of a Nikon microscope (Nikon, Japan). The chamber was continuously superfused (~1.0 ml/min) with Tyrode solution. The whole-cell configuration of the patch-clamp technique was applied. Briefly, glass electrodes (World Precision Instruments, Sarasota, FL, USA) with 1 to 3 MΩ resistance were connected via an Ag-AgCl wire to an Axopatch 200A amplifier interfaced with a DigiData-1320 acquisition system (Molecular Devices, Inc., Sunnyvale, CA, USA). After forming a conventional "gigaohm" seal, electrode capacitance was compensated. Additional suction ruptured the patched membrane and formed the whole-cell configuration. Cell membrane capacitance (C_m_) was routinely recorded from each patched cell with the pCLAMP program (version 9.2, Molecular Devices, Inc., Sunnyvale, CA, USA). Ca^2+ ^currents were recorded with the protocols similar to those previously described[25]. Action potential (AP) was evoked and recorded with the whole-cell current-clamp method[26].

### Chemicals and solutions

GLP-1 and its receptor inhibitor Exendin (9-39) were purchased from Bachem Americas, Inc. (Torrance, CA, USA). GLP-1 (7-36 amide) was tested at concentrations ranging from 0.05 nM to 5 nM for its effect on isolated canine left ventricular myocytes. The effects of Exendin (9-39) or the cAMP-dependent protein kinase A (PKA) inhibitor 4-25 fragment (Sigma, St. Louis, MO, USA) were also assessed in isolated single cardiomyocytes with or without GLP-1 treatment.

For recording Ca^2+ ^currents, the bath solution contained (in mM): NaCl 140, CsCl 5, MgCl_2 _1, CaCl_2 _1.8, glucose 5, and HEPES 10 (pH 7.4 with HCl). The pipette solution contained (in mM): CsCl 100, CsOH 40, MgCl_2 _1, CaCl_2 _1, EGTA 11, Mg-ATP 5, and HEPES 10 (pH 7.3 with CsOH). ATP and all other chemicals used in this study were obtained from Sigma Aldrich (St. Louis, MO, USA). The bath solution for recording APs contained (mM): NaCl 140; KCl 5.4; CaCl_2 _1.8; MgCl_2 _1; D-glucose 5; HEPES 10 (pH adjusted to 7.4 with NaOH). The pipette solution for recording APs contained (mM): K-glutamate 130; KCl 15; NaCl 5; MgATP 5; MgCl_2 _1; EGTA 5; CaCl_2 _1; HEPES 10 (pH adjusted to 7.2 with KOH). A perfusion system was used to change the extracellular solution. Data were collected with pCLAMP (version 9.2, Axon Instruments, CA, USA). All experiments were conducted at room temperature (~22°C).

### Data analysis

Peak I_Ca _was measured in the presence or absence of GLP-1 or other chemical compounds. The parameters of APs were analyzed by using the methods similar to those described in a previous report[26]. The maximal effects of GLP-1 on I_Ca _and APs were used in the table and figures. All data were presented as mean ± standard error of the mean unless otherwise stated. The Student's *t*-test and ANOVA were applied for statistical analysis as appropriate. Differences were considered significant if *p *< 0.05.

## Results

### Effects of GLP-1 and Exendin (9-39) on L-type Ca^2+ ^currents

**Figure 1:**
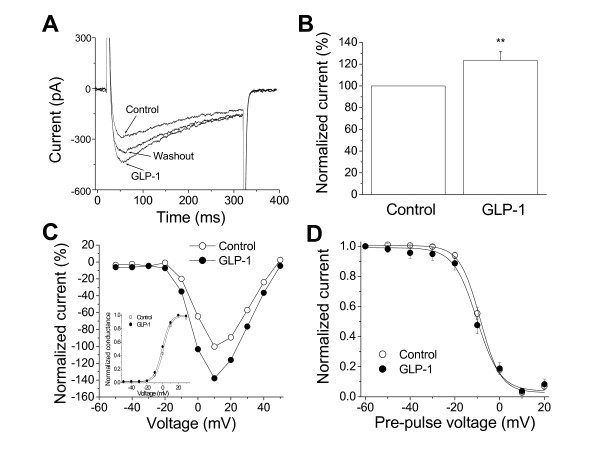
**Effects of GLP-1 on voltage-gated L-type Ca**^**2+ **^**currents in isolated canine left ventricular myocytes**. The representative traces of I_Ca _in the absence (Control and Washout) and presence (GLP-1) of 5 nM GLP-1 are shown in panel ***A***. The currents were evoked by the depolarizing pulses from the holding potential of -40 mV to 0 mV every 10 s. The current traces represented the I_Ca _recorded at the times just before GLP-1 (Control), 12 min after GLP-1 perfusion (GLP-1), and 10 min after washout of GLP-1 (Washout). Panel ***B ***shows the average increase in the peak I_Ca _in the presence of 5 nM GLP-1 (n = 7, *p *< 0.01). Panel ***C ***shows the effects of 5 nM GLP-1 on the current-voltage relationship of I_Ca _recorded from a representative myocyte in the presence (solid circle) and absence (open circle) of 5 nM GLP-1. The normalized currents were calculated as the ratio of the peak I_Ca,GLP-1 _to the maximal peak I_Ca,Control_. The voltage protocol was composed of a group of pulses from -50 mV to 50 mV with 10 mV increments every 10 s. The membrane holding potential was -40 mV. The inset in the panel ***C ***shows the activation curves in the presence (solid circle) and absence (open circle) of 5 nM GLP-1. Panel ***D ***shows the steady-state inactivation of L-type Ca^2+ ^currents (n = 7) in the presence (solid circle) and absence (open circle) of 5 nM GLP-1. The voltage protocol had double pulses consisting of a 200-ms test pulse to 0 mV following a 500-ms conditioning pulse varying from -60 to 20 mV in 10-mV increments every 10 ms with membrane holding potential of -40 mV. Normalized inactivation data were fit to a Boltzmann equation (solid lines): *y *= 1/{1 + exp[(V + V_0.5_)/*K*]}, where V_0.5 _is the voltage at which *y *= 0.5 and *K *is the slope factor.

Extracellular perfusion of 5 nM GLP-1 (7-36 amide) solution gradually increased the peak currents and reached the maximal effects around 10 to 15 min. Figure 1A shows the representative traces in the presence or absence of 5 nM GLP-1. The enhancement effect of GLP-1 on I_Ca _was washable upon washing away of the compound. While GLP-1 at 5 nM significantly increased the peak currents (Fig. 1B), the current-voltage relationship of the activation was not altered and the kinetics of the activation and the steady-state inactivation of I_Ca _slightly shifted, but not significantly, to the hyperpolarizing direction in the presence of 5 nM GLP-1 (Fig. 1C and 1D, n = 7).

**Figure 2:**
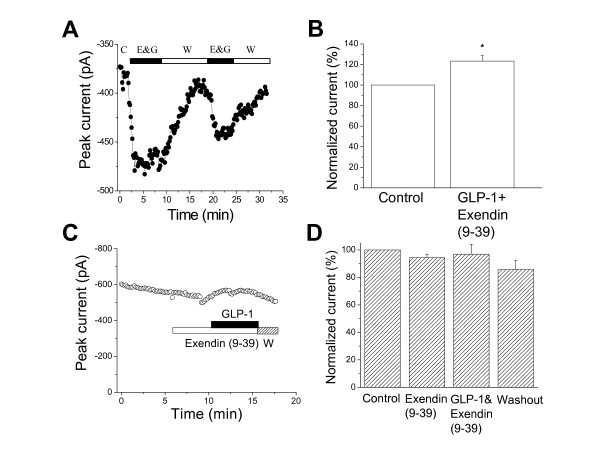
**Effects of the GLP-1R inhibitor Exendin (9-39) on the GLP-1-induced enhancement of L-type Ca**^**2+ **^**currents in isolated canine left ventricular myocytes**. ***A***, The time course of the extracellular perfusion of Exendin (9-39, 100 nM) plus GLP-1 (5 nM) is shown. The GLP-1-induced enhancement of Ca^2+ ^currents was not blocked by Exendin (9-39), even after washout and reperfusion of these two compounds. The currents were evoked by the depolarizing pulses from a holding potential of -40 mV to 0 mV every 10 s. C, control; E&G, Exendin (9-39) + GLP-1; W, washout. ***B***, Compared to control, the average increase in peak I_Ca _was statistically significant in the presence of Exendin (9-39) plus GLP-1 (n = 6, *p *< 0.05). Panel ***C ***shows the time course of I_Ca _for control, the extracellular perfusion of 100 nM Exendin (9-39) alone (Exendin (9-39)) and Exendin (9-39) plus 5 nM GLP-1 (GLP-1), and washout (W). The currents were evoked by the pulses depolarizing from holding potential of -40 mV to 0 mV every 10 s. ***D***, The averaged data show that 100 nM Exendin (9-39) applied 5 min ahead of GLP-1 (5 nM) perfusion abolished the GLP-1-induced enhancement of I_Ca _(n = 5, *p *> 0.05).

To determine whether the GLP-1-induced enhancement of I_Ca _was via the activation of membrane GLP-1Rs, the GLP-1R inhibitor Exendin (9-39) was added to the GLP-1 solution. The non-mammalian peptide, Exendin (9-39 amide), is a specific and competitive antagonist of GLP-1Rs. As Exendin (9-39) has lower binding affinity to GLP-1Rs, 100 nM Exendin (9-39), at a concentration of 20 times higher than that of GLP-1, plus 5 nM GLP-1, was perfused to the canine left ventricular myocytes. Interestingly, Exendin (9-39) at 100 nM was unable to block the GLP-1-induced enhancement of I_Ca _(Fig. 2A and 2B). These results suggest that when GLP-1 and Exendin (9-39) were applied together at the same time, GLP-1 could activate GLP-1Rs due to its higher affinity for GLP-1 receptors. After the activation of GLP-1Rs, the enhancement of I_Ca _by GLP-1 was not blocked by 100 nM Exendin (9-39) because the activation process was already beyond the receptor stage. To test this hypothesis, we administered 100 nM Exendin (9-39) 5 min prior to GLP-1. Figure 2C and 2D clearly shows that Exendin (9-39) alone did not significantly alter the currents, but the enhancement of I_Ca _by 5 nM GLP-1 was abolished by pre-administration of 100 nM Exendin (9-39).

### Protein kinase A and GLP-1-induced enhancement of Ca^2+ ^currents

**Figure 3:**
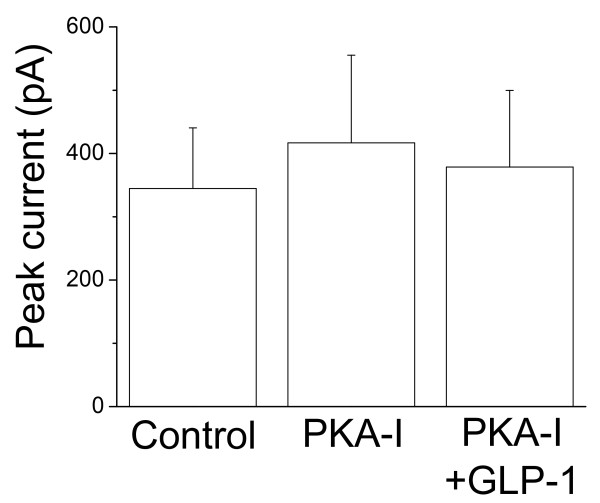
**Intracellular dialysis of PKA-I (PKA-I fragment 4-25, 5 μM) prevented the GLP-1-induced enhancement of I**_**Ca **_**in isolated canine left ventricular myocytes (n = 5)**. Ca^2+ ^currents were evoked by the depolarizing pulses from -40 mV to 0 mV every 10 s. Control, I_Ca _was recorded immediately after forming the whole-cell configuration; PKA-I, I_Ca _was recorded at 20 min after forming the whole-cell configuration and PKA-I dialysis; PKA-I+GLP-1, I_Ca _was recorded at 35 min after PKA-I dialysis and 15 min after GLP-1 perfusion.

To determine whether the GLP-1-induced enhancement of I_Ca _was via activation of the cAMP/PKA pathway, the PKA inhibitor (PKA-I fragment 4-25 amide, 5 μM) was added to the electrode solution. After forming a whole-cell configuration, I_Ca _was immediately recorded (Fig. 3, Control). Each cell was dialyzed for 20 min with PKA-I and I_Ca _was recorded. The peak currents recorded at 20 min after intracellular PKA-I dialysis were not significantly changed (Fig. 3, PKA-I). Extracellular perfusion of 5 nM GLP-1 for 15 min did not induce a significant increase in I_Ca _(Fig. 3, PKA-I+GLP-1). Compared with the significant increase in I_Ca _of the cardiomyocytes treated with 5 nM GLP-1 alone (23 ± 8%, n = 7, *p *< 0.05, Fig. 1B), I_Ca _in the myocytes with intracellular dialysis of PKA-I was not significantly altered by the same concentration of GLP-1 (8 ± 13%, n = 5, *p *> 0.05, Fig. 3, PKA-I+GLP-1). This result suggests that GLP-1-induced enhancement of I_Ca _was most likely via the activation of the PKA pathway.

### GLP-1-induced prolongation of action potential duration

**Figure 4:**
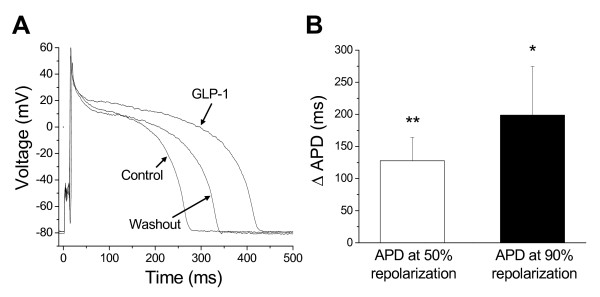
**Effects of GLP-1 on action potentials in isolated canine left ventricular cardiomyocytes**. ***A***, Extracellular perfusion of 5 nM GLP-1 (GLP-1) prolonged the action potential duration and the effect was removable after washing out of GLP-1 (Washout). ***B***, GLP-1 at 5 nM prolonged the action potential durations measured at 50% (APD50) and 90% (APD90) repolarization. ΔAPD, the differences between control and 5 nM GLP-1. *, *p *< 0.05; **, *p *< 0.01; versus control.

As L-type Ca^2+ ^currents can affect the plateau of a cardiac action potential (AP) and as GLP-1 could enhance I_Ca_, we next investigated the effects of GLP-1 on the AP duration (APD). Figure 4A shows that extracellular perfusion of 5 nM GLP-1 gradually prolonged the APDs of the patched cardiomyocyte. The prolongation effect was initiated within 2 min after GLP-1 perfusion and reached the maximal level around 10 min. The GLP-1-induced APD prolongation was recoverable after washing out of the compound (Fig. 4A and Table 1). The durations of the APs measured at 50% and 90% repolarization were significantly prolonged in the presence of 5 nM GLP-1 (Fig. 4B and Table 1). Other AP parameters, such as amplitude, threshold, and maximum upstroke velocity of AP, were not significantly altered in the presence of 5 nM GLP-1. The APD prolongations showed a concentration-dependent trend. The delta prolongations were 24.8 ± 11.5 ms and 25.7 ± 15.7 ms for 0.05 nM GLP-1, and 42.2 ± 25.1 ms and 48.5 ± 25.2 ms for 0.5 nM GLP-1 measured at 50% and 90% repolarization, respectively, but these changes did not reach statistical significance at both concentrations (*p *> 0.05, n = 6).

**Table 1:** Effects of GLP-1 on action potentials of canine left ventricular cardiomyocytes

	MHP mV	AAP mV	APT mV	Vmax V/s	**APD**_**50 **_**ms**	**APD**_**90 **_**ms**
Control	-80.2 ± 1.9	149.8 ± 4.6	-61.0 ± 2.4	126.6 ± 17.6	124.3 ± 24.4	236.1 ± 17.6
GLP-1	-84.2 ± 2.0	145.3 ± 4.5	-61.8 ± 1.6	120.8 ± 22.7	252.1 ± 30.5**	434.8 ± 79.2*
Washout	-82.4 ± 2.3	142.2 ± 5.8	-62.4 ± 0.9	113.1 ± 25.5	120 ± 27..7	302.8 ± 66.9

Values are expressed as mean ± SEM (n = 6). GLP-1 (7-36 amide) at 5 nM was added to the bath solution. The data were collected at the times just before GLP-1 (Control), 10 to 15 min after GLP-1 perfusion (GLP-1), and 10 to 15 min after washout of GLP-1 (Washout). MHP, membrane holding potential before elicitation of an action potential; AAP, amplitude of action potential; APT, threshold of initiation of an action potential; V_max_, maximum upstroke velocity of action potential; APD, action potential duration measured at 50% (APD_50_) and 90% (APD_90_) of repolarization. *, *p *< 0.05; **, *p *< 0.01; versus control and washout.

## Discussion

The main finding in this study is that GLP-1 (7-36 amide) can enhance voltage-gated L-type Ca^2+ ^currents in isolated canine left ventricular myocytes. The GLP-1-induced enhancement of Ca^2+ ^currents was significantly attenuated in the presence of the GLP-1R inhibitor Exendin (9-39) (Fig. 2) or by intracellular dialysis with the PKA inhibitor, PKA-I fragment 4-25 (Fig. 3). The effect of GLP-1 on I_Ca _observed in this study may help to interpret the chronotropic effect of GLP-1 *in vivo*[8-10], perhaps via the modulation of Ca^2+ ^channels of sinoatrial node cells.

### GLP-1 receptors in cardiomyocytes

The existence of GLP-1Rs has been demonstrated in many human organs and tissues, including the pancreas, heart, lung, kidney, stomach and brain [5]. The data from the current study suggest that canine left ventricular myocytes functionally express GLP-1Rs, because GLP-1 enhanced I_Ca _and prolonged APD, and also because the GLP-1-induced enhancement of I_Ca _was blocked by specific GLP-1R inhibitor Exendin (9-39). However, the concentration of Exendin (9-39) required to block the GLP-1 effects was relatively high, and the antagonist was also required prior to administration of GLP-1 (Fig. 2). This suggests that compared with GLP-1, Exendin (9-39) is a competitive inhibitor with a lower GLP-1R binding affinity and slower binding kinetics at the cardiac GLP-1R. There is a possibility that the effects of GLP-1 on myocyte electrophysiology is via a GLP-1R-independent mechanism. However, since no report so far has shown that GLP-1 can affect cellular function via a non-receptor approach, and since pre-administration of Exendin (9-39) abolished the increase in I_Ca _subsequent to GLP-1 administration, we believe that a receptor-independent mechanism of the GLP-1-induced enhancement of I_Ca _is most unlikely.

### GLP-1 and the cAMP/PKA pathway

Voltage-gated L-type Ca^2+ ^currents influence the morphology of cardiac action potential, especially the plateau phase, and are also critical for excitation-contraction coupling in the heart. Stimulation of the cardiac β-adrenergic receptor increases Ca^2+ ^influx through the voltage-gated L-type Ca^2+ ^channel[27]. The cardiac β-adrenergic receptor is coupled to an intracellular signaling cascade via the stimulatory G protein (G_s_) which can activate the cAMP-dependent PKA system and enhance Ca^2+ ^channel phosphorylation. In cardiomyocytes, the PKA-dependent phosphorylation of L-type Ca^2+ ^channels increases I_Ca_[27-29]. In the current study, we found that the GLP-1-induced increase in I_Ca _was abolished by intracellular dialysis of a PKA inhibitor (PKA-I fragment 4-25). This result suggests that GLP-1 activates the GLP-1R which couples with the G-protein/adenyl cyclase complex to enhance cAMP production. Increase in intracellular cAMP activates PKA and then enhances Ca^2+ ^channel phosphorylation. Phosphorylated Ca^2+ ^channels increase their open probability when they are activated by voltage change. This is why the GLP-1-induced enhancement of I_Ca _was blocked by PKA-I. These results are along with the findings in the previous studies in rodent cardiomyocytes that GLP-1 or the GLP-1R agonist liraglutide significantly increased intracellular cAMP, which was abolished by the GLP-1R antagonist Exendin (9-39)[13,30]. Also, in the pancreatic β-cell, GLP-1 enhances cytoplasmic Ca^2+ ^oscillation and insulin secretion via activation of cAMP cascades[22,23].

The effects of GLP-1 on I_Ca _are most likely via the activation of the GLP-1R and then the cAMP-dependent PKA pathway. Activation of the PKA pathway is widely recognized as a key intracellular signaling mechanism in ischemic and pharmacological preconditioning. Subsequent cardioprotection of the PKA system may be beneficial in both treating/preventing ischemic damage, as well as apoptosis in heart failure. In studies by Bose et al, it has been shown that GLP-1 administration can reduce ischemia-reperfusion injury in part via activation of PKA, as well as via activation of other pro-survival kinases, and can be blocked by PKA inhibitors and inhibitors of pro-survival kinases[12,31,32]. One of the limitations in our experiments is that we did not directly measure intracellular cAMP contents with or without GLP-1 or GLP-1 plus a blocker of GLP-1R. However, one previous study has shown that GLP-1 at 10 nM doubled intracellular cAMP content in rat cardiac myocytes[13]. The GLP-1R agonist liraglutide at 100 nM significantly increased the level of intracellular cAMP in mouse cardiomyocytes and this increase was abolished by the GLP-1R antagonist Exendin (9-39)[30]. In addition, GLP-1 exerts cAMP/PKA-mediated insulinotropic actions in endocrine tissues[19,33,34] and stimulates adenylate cyclase to cause an increase in cAMP in pancreatic islet cells[34,35]. These results strongly suggest that the cellular effects of GLP-1 involve the cAMP-dependent PKA pathway and our data are consistent with the previous findings.

The GLP-1-induced enhancement of I_Ca _can trigger intracellular Ca^2+ ^release. Elevation of intracellular Ca^2+ ^level can lead to an improvement of myocardial contractility. Several studies in pancreatic β-cells have shown that GLP-1 synchronizes Ca^2+ ^and cAMP oscillations[10,19,36] and such oscillations involves the ryanodine-sensitive Ca^2+ ^store[22]. In addition, the experimental evidence has shown that GLP-1 can augment Ca^2+ ^influx[37,38] and Ba^2+ ^currents[39] through L-type Ca^2+ ^channels via the cAMP-dependent PKA pathway in pancreatic β-cells. In addition, GLP-1 increases intracellular cAMP content in adult rat cardiomyocytes[13].

### Pharmacological effects of GLP-1

Our data demonstrate that GLP-1 can modulate cardiomyocyte electrophysiology. However, GLP-1 (7-36 amide) in human plasma is normally less than 100 pM[40-42]. GLP-1 administration at 5 nM to obtain a significant effect on APs and I_Ca _in this study is much higher than a physiological level. Therefore, the effects of GLP-1 on I_Ca _and APs are most likely pharmacological in this study. Various pharmacological concentrations ranging from 10 to 100 nM of GLP-1 or GLP-1R agonists have been tested in several previous studies. In one clinical trial, GLP-1 (17 nmol) was infused to assess its antidiabetogenic action[40]. GLP-1 (up to 100 nmol/kg) was used to test its effects on insulin secretion, insulin sensitivity, and glucose effectiveness in mice[43]. Moreover, 0.1 to 1000 nM GLP-1 was used to assess the dose-dependent effect of GLP-1 on cAMP production with an EC_50 _of 10 nM in rat cardiomyocytes[13] and 50 nM GLP-1 to study the role of L-type Ca^2+ ^channels in mediating GLP-1-stimulated events in cultured pancreatic β-cell line INS-1[44]. As the GLP-1R agonists, Byetta™ and Victoza™, have been used for the treatment of patients with type II diabetes mellitus, the concentration of GLP-1 used in this study can have pharmacological significance.

The beneficial effects of GLP-1 at various pharmacological concentrations have been shown in several recent animal studies. Chronic treatment with either GLP-1 or AC3174, a peptide analogue with pharmacologic properties similar to the GLP-1R agonist exenatide, showed cardioprotective effects and improved cardiac function, cardiac remodeling, insulin sensitivity, and exercise capacity in myocardial infarction rats with chronic heart failure[45]. Chronic AC3174 treatment also attenuated salt-induced hypertension, cardiac morbidity, insulin resistance, and renal dysfunction and improved survival in Dahl salt-sensitive rats[46]. In addition, GLP-1 has an antiapoptotic effect on β-cells during oxidative stress probably via blocking the c-Jun-N-terminal kinase (JNK) and glycogen synthasekinase 3β (GSK3β) mediated apoptotic pathway[47]. So far, GLP-1 and its analogs are only available as injectable dosage forms. Recently, a single dose of the exenatide-based ORMD-0901 formulation was enterically delivered to pigs and beagle canines[48]. The results show that enterically delivered ORMD-0901 was well tolerated by the animals and that GLP-1 (ORMD-0901) was absorbed from the gastrointestinal tracts and retained its biological activity. Hence, development of this drug class in an oral dosage form has the potential to enhance diabetes control and patient compliance.

## Conclusions

Our data demonstrate for the first time that GLP-1 (7-36 amide) can enhance voltage-gated L-type Ca^2+ ^currents in cardiomyocytes and such enhancement may contribute to the prolongation of APs. These effects are most likely via the activation of the cardiac GLP-1R and then the cAMP-dependent PKA pathway. These findings suggest a novel mechanism of the potential beneficial cardiac effects of GLP-1R agonists on the improvement of myocardial contractility.

## Abbreviations

AP: action potential; APD: action potential duration; cAMP: 3'-5'-cyclic adenosine monophosphate; GLP-1: glucagon-like peptide-1; GLP-1R: glucagon-like peptide-1 receptor; I_Ca_: voltage-gated L-type Ca^2+ ^current; PKA: protein kinase A; PKA-I: protein kinase A inhibitor

## Competing interests

All authors were employees and held stock in Medtronic, Inc at the time these experiments were performed.

## Authors' contributions

YFX designed the study, conducted the patch clamp experiments, analyzed and interpreted the results, wrote the manuscript. AN collected canine heart tissues and isolated the ventricular cardiomyocytes. AN, DAJ, and DCS participated the discussion of experimental design and manuscript writing and editing. All authors read and approved the final manuscript.

## Authors' information

YFX is a principal scientist at Medtronic, Inc. and an adjunct professor at the Department of Pharmacology and Physiology, New Jersey Medical School, University of Medicine and Dentistry of New Jersey, NJ, USA. AN was a research scientist at Medtronic, Inc. DAJ is a senior scientist at Medtronic, Inc. DCS was a senior research manager at Medtronic, Inc. and is currently an adjunct assistant professor at the Department of Integrative Biology and Physiology, University of Minnesota, MN, USA.
